# Interactions between *Bacillus anthracis* and Plants May Promote Anthrax Transmission

**DOI:** 10.1371/journal.pntd.0002903

**Published:** 2014-06-05

**Authors:** Holly H. Ganz, Wendy C. Turner, Eoin L. Brodie, Martina Kusters, Ying Shi, Heniritha Sibanda, Tamas Torok, Wayne M. Getz

**Affiliations:** 1 Department of Environmental Science, Policy and Management, University of California, Berkeley, Berkeley, California, United States of America; 2 Ecology Department, Earth Science Division, Lawrence Berkeley National Laboratory, Berkeley, California, United States of America; 3 Independent researcher, Windhoek, Namibia; 4 Department of Statistics, University of California, Berkeley, California, United States of America; 5 Ministry of Fisheries and Marine Resources, Inland Aquaculture, Katima Mulilo Regional Office, Katima Mulilo, Namibia; 6 School of Mathematical Sciences, University of KwaZulu-Natal, Durban, South Africa; University of California San Diego School of Medicine, United States of America

## Abstract

Environmental reservoirs are essential in the maintenance and transmission of anthrax but are poorly characterized. The anthrax agent, *Bacillus anthracis* was long considered an obligate pathogen that is dormant and passively transmitted in the environment. However, a growing number of laboratory studies indicate that, like some of its close relatives, *B. anthracis* has some activity outside of its vertebrate hosts. Here we show in the field that *B. anthracis* has significant interactions with a grass that could promote anthrax spore transmission to grazing hosts. Using a local, virulent strain of *B. anthracis*, we performed a field experiment in an enclosure within a grassland savanna. We found that *B. anthracis* increased the rate of establishment of a native grass (*Enneapogon desvauxii*) by 50% and that grass seeds exposed to blood reached heights that were 45% taller than controls. Further we detected significant effects of *E. desvauxii, B. anthracis*, and their interaction on soil bacterial taxa richness and community composition. We did not find any evidence for multiplication or increased longevity of *B. anthracis* in bulk soil associated with grass compared to controls. Instead interactions between *B. anthracis* and plants may result in increased host grazing and subsequently increased transmission to hosts.

## Author Summary

Anthrax is a neglected zoonotic disease affecting livestock, wildlife, and humans in developing countries, particularly in Africa and Asia, and it occurs regularly in rural parts of North America. The causative agent of anthrax, *Bacillus anthracis* is transmitted by spores that persist for long periods of time in the environment. The transmission mechanisms of socioeconomically important and environmentally maintained pathogens are poorly understood, yet essential for understanding disease dynamics and devising appropriate control measures. Recent laboratory studies show that *B. anthracis* interacts with plants and other soil-dwelling organisms that may affect its survival and transmission. In this paper, we describe the results of a field experiment designed to test whether the interaction of *B. anthracis* with plants might affect its persistence and potential transmission to grazing hosts. We found that like some of its close relatives, *B. anthracis* promotes plant growth. Rather than simply lying in wait as a dormant spore in soil, instead *B. anthracis* may promote plant growth as a way of attracting hosts to graze on infectious material at carcass sites.

## Introduction

The causative agent of anthrax in humans, livestock and wildlife, *Bacillus anthracis* was long considered an obligate pathogen that is dormant in the environment [1], [2]. This view was supported by early laboratory studies conducted by Minett and Dhanda [3] and Minnet [4] that suggest that *B. anthracis* is unable to compete with other soil bacteria. However, a growing number of laboratory studies indicate that, like some of its close relatives, *B. anthracis* may interact with other members of grassland communities, including plants [5], earthworms [6], flies [7], and soil amoeba [8]. *Bacillus anthracis* belongs to the closely related *Bacillus cereus* sensu lato, which contains both obligate and opportunistic animal pathogens, including *B. cereus* sensu stricto and *Bacillus thuringiensis* [9]. A common soil saprobiont [10], *B. cereus* causes opportunistic infections in humans [11] and lives symbiotically in invertebrate guts [12], [13]. An insect pathogen commonly used as a pesticide in agriculture [14], *B. thuringiensis* also occurs in soil and can be found living symbiotically in caterpillar guts [9].

In his pioneering research, Pasteur proposed that earthworms vector *B. anthracis* from buried livestock carcasses [15], [16]. Following up on these observations, Schuch and Fischetti [6] found that bacteriophages generate phenotypic changes in *B. anthracis* that enable it to persist as an endosymbiont in earthworms and to act as a saprobiont in soil and water. In a laboratory study, Saile and Koehler [5] demonstrated that *B. anthracis* germinates, survives as a saprobiont and undergoes horizontal gene transfer in the plant rhizosphere. Significantly, Dey et al. [8] showed that the virulent Ames strain of *B. anthracis* germinates and multiplies intracellularly within a free-living soil amoeba living in moist soils and that the pXO1 plasmid was essential for growth.

Transmission of *B. anthracis* is environmentally mediated and such ecological interactions in the environment likely affect its transmission to subsequent hosts that contact and ingest spores while grazing [17]. In the late 19^th^ century, Louis Pasteur identified carcass sites as a key area for anthrax transmission [15]. At these locations, after a vertebrate host has succumbed to an anthrax infection, vegetative cells of *B. anthracis* are released (along with blood and other body fluids) into the environment and produce infectious spores capable of long-term survival [3], [18]–[20]. The carcass also provides a pulse of nutrients into the soil, promoting plant growth [21]–[23] and supporting *B. anthracis* survival and replication [3], [4]. In addition to nutrients, the inoculation of *B. anthracis* into carcass site soils may also promote the growth of plants. In arid or semiarid areas, plants inoculated with beneficial bacteria grow better and are more drought-tolerant [24], [25]. Some *Bacillus* spp., including *B. thuringiensis* help plants survive drought conditions through effects on osmoregulation and antioxidant activity [26]. If it occurs, an association between *B. anthracis* and plants may also provide a potential route of transmission to grazing hosts.

**Figure 1:**
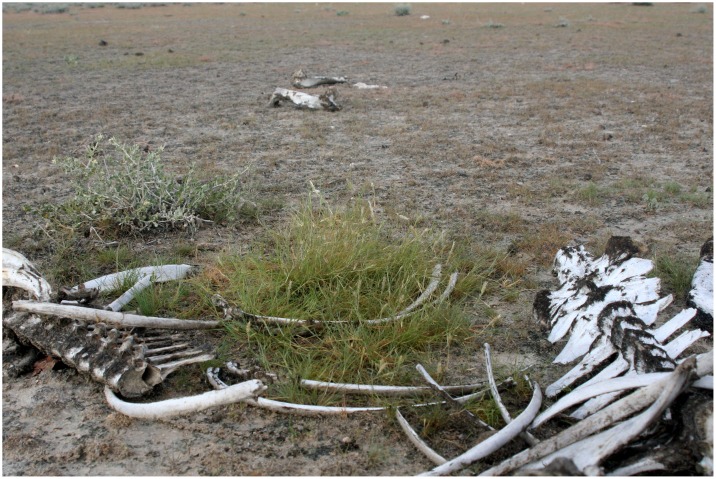
The grass, *Enneapogon desvauxii* growing near the bones of a plains zebra that died from anthrax one year previously in Etosha National Park, Namibia.

We performed a field experiment designed to test whether interactions between a local virulent strain of *B. anthracis*, a native grass, and blood might promote *B. anthracis* survival and the likelihood of its transmission in the environment. For this study, we selected a species of grass, *Enneapogon desvauxii*, a pioneer species that tolerates overgrazing and occurs in the dry calcareous soils [27] characteristic of our study site in Etosha National Park (Etosha), Namibia. As part of another study on the epidemiology of anthrax, we noted the growth of *E. desvauxii* and other grasses near sites where animals had died previously from anthrax (Figure 1). *Enneapogon desvauxii* is also a preferred forage for plains zebra (*Equus quagga*) [28], [29], the most common victim of anthrax infections in Etosha [30]. This research was conducted in a semi-arid dwarf shrub savanna [31] in Etosha, a large (22,915 km^2^) reserve with abundant wildlife populations that exhibit regular occurrences of anthrax infections (reviewed in [30]). These plains support a high density of anthrax-susceptible herbivores, including plains zebra, blue wildebeest (*Connochaetes taurinus*), springbok (*Antidorcas marsupialis*), and gemsbok (*Oryx gazella*). We tested the following hypotheses in a manipulative field experiment:

1. Carcass materials (*B. anthracis* spores and zebra blood) promote *E. desvauxii* growth.
2. *E. desvauxii* increases the persistence of *B. anthracis* in soil.
3. *E. desvauxii* (or *B. anthracis*) alters the structure of soil bacterial communities.

## Materials and Methods

### Ethics statement

Field research was authorized by the Namibian Ministry of Environment and Tourism (MET) under the auspices of Permit Number 1220/2007 to Wayne M. Getz. The sample collection protocol was approved by the University of California, Berkeley (UCB) Animal Care and Use Committee (ACUC, Animal Use Protocol #R217-0509B). Further, we followed all conditions set forth in a Biological Use Authorization permit approved by the UCB Committee on Laboratory and Environmental Biosafety and the UCB ACUC.

### Study site

**Figure 2:**
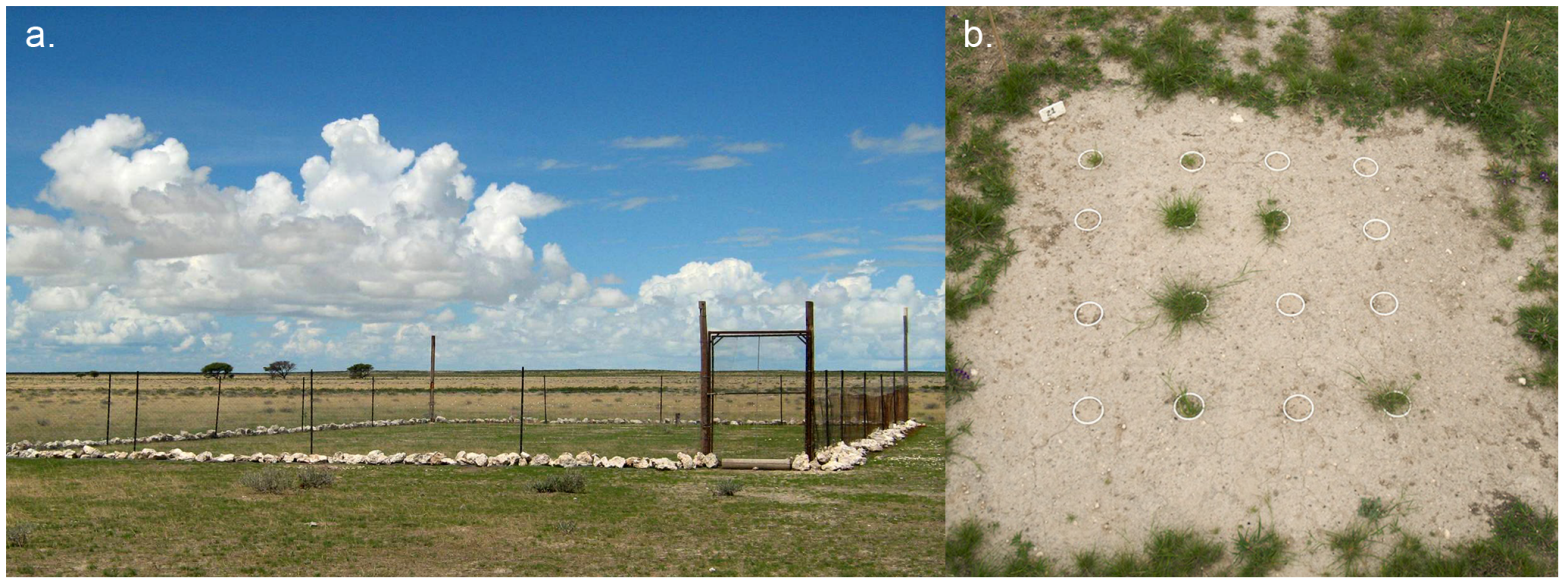
Experimental design: a. The fenced field enclosure situated in the grassland savanna in central Etosha. b. One of twenty-five 1 m^2^ plots established within the field enclosure. Each plot contained two replicates for each of eight treatments.

In late October 2008, we established a fenced area (26.5 m long×15.5 m wide×1.4 m high) in the savanna, 4.8 km north of Leeubron in central Etosha, in order to restrict access by grazing animals and scavengers (Figure 2a). We characterized physical and chemical parameters in five soil core samples (5 cm diameter ×10 cm depth) collected from five randomly selected locations throughout the site (Table S1), following the methods of Ganz et al. [32]. Soil samples from the field enclosure had a pH of 7.8±0.04 (±SE), had a sandy, loamy texture and exhibited some variation in sodium content, electrical conductivity (consistent with salt deposits), and particle size (clay and silt content, Table S1). Soils in this part of Etosha were previously described as dominated by shallow to medium, weakly developed, carbonate-rich, silty loamy to sandy-loamy Regosols and Leptosols from mainly aeolian origin that cover a limestone surface [33].

To ensure that we did not establish our experimental site in a previously contaminated area, we tested for *B. anthracis* by culturing five soil core samples using polymyxin-lysozyme-EDTA-thallous acetate (PLET) agar as described below. In addition, we extracted DNA and performed PCR for a *B. anthracis* specific marker as described below. We did not find any evidence for *B. anthracis* cells or DNA present in the experimental enclosure prior to the study. DNA was extracted from 10 g of soil using the PowerMax Soil DNA extraction kit (MoBio, Carlsbad, CA, USA). DNA was concentrated by isopropanol precipitation and quantified using the Picogreen Assay (Invitrogen, Carlsbad, CA, USA) on a NanoDrop 3300 fluorometer (Thermo Scientific, Wilmington, DE). We performed PCR for a 160 bp diagnostic chromosomal region for *B. anthracis*, using specific primers (BA177F: TTGGATCAGCGTTTCTGAATTCAGC and BA177R: TCCCCATATCGCTCAATTCCATCTA) [34]. The PCR protocol was as follows: initial denaturation at 95°C for 3 min, 35 cycles of denaturation at 95°C for 30 s, annealing at 60°C for 30 s and extension at 72°C for 30 s, and a final extension at 72°C for 7 min. The products were visualized on 2% agarose gels in 1% Tris-borate-EDTA. In addition, we performed a second PCR with the products from the first reactions.

### Experimental design

Within the enclosure, we conducted a full factorial experiment: 2 grass treatments (*E. desvauxii* seedling present or absent) ×2 spore treatments (*B. anthracis* spores present or absent) ×2 blood treatments (blood present or absent), for a total of eight treatments. In each of 25 plots (1 m^2^), we distributed sections of 5 cm diameter PVC pipe (10 cm length) in a 4×4 grid and hammered the pipe sections into the soil (Figure 2b). Each section of pipe was placed at least 15 cm apart and the surface of each pipe section extended about 0.5 cm above the ground. Each plot was divided in half and every treatment was randomly assigned to one sample unit within each subplot. In the plant treatments, 20 locally collected seeds of *E. desvauxii* were placed under about 2 cm soil in the PVC pipes. Seeds were allowed to germinate naturally. At each *B. anthracis* present site, we dispensed 100 million spores in 1 ml distilled water onto the surface soil inside each PVC pipe that was flush with the soil surface and we placed the same volume of sterile distilled water in each of the other treatments at the same time to control for the effects of water.

We placed 1.5 ml zebra blood at each replicate of the blood treatment and we placed the same volume of sterile distilled water in the other treatments. The blood was collected from anesthetized zebras (*E. quagga*) in 10 ml tubes containing 143 units of sodium heparin to prevent coagulation. All blood was pooled prior to dispensing in the experimental plots.

### Preparation of spore treatment

The *B. anthracis* isolate used in this study was obtained from a swab of a zebra carcass from 2006 (Etosha Ecological Institute (EEI) carcass number: EB060318-01WV, GPS coordinates: −18.99736, 15.81584). This isolate was identified as genotype 6 in the A cluster of *B. anthracis* [35]. Genotype 6 belongs to a dominant *B. anthracis* strain that has been causing outbreaks in the ENP for at least the past 25 years and is the ancestor of all genotypes currently found there [35].

Spores were grown to spike into the soil for the experiment by streaking the isolate onto a Tryptone Bile Agar plate. Following an overnight incubation at 30°C, we used a 1 µl loop to suspend a colony in 2 ml sterile water and we spread 100 µl of the resulting cell suspension onto 2× SG agar (modified Schaeffer's sporulation agar, [36]). Plates were incubated at 37°C for 48 h. When sporulation efficiency was determined by microscopy to have reached nearly 100%, the cell lawn was harvested and suspended in 30 ml cold sterile water. Cells were collected by centrifugation for 20 min at 3,000 *g* and washed three times with cold sterile distilled water. We applied a step-density gradient using Reno-Cal-76 in order to separate the spores from vegetative cells. Each pellet was centrifuged at 3,000 g for at least 60 min. The supernatant was removed by aspiration. The pellet was washed with 1 ml cold sterile distilled water three times. The spores were suspended in sterile distilled water and diluted to 100 million spores per ml based on microscopic counts using a counting chamber. One homogenized ml of this spore preparation was used for the spore present treatment.

### Plant measurements

The grass began to emerge after the rains began in January 2009. We recorded the percentage of experimental units where at least one *E. desvauxii* seedling emerged by late January 2009. At the end of the growing season (in late March 2009), we collected nine replicates for each of the four plant treatments (control, spore, blood, spore and blood) and measured maximum stem height (including inflorescences) and root length up to a maximum depth of 10 cm. We recorded seedling establishment and plant growth in the first year only for two main reasons. First, a wildfire swept rapidly through the field enclosure on October 10^th^ 2009 singeing the grass unevenly. Second, as the experiment progressed, it became difficult to distinguish the growth of new plants that established from seeds in *E. desvauxii* from prior growth (including roots) [27].

### Measuring *B. anthracis* total counts

We removed three replicates from each treatment at four different time points from January 2009 until April 2011, which occurred during the Namibian rainy season. We sampled on January 27^th^ 2009 after the grass seedlings emerged. We collected samples in the late rainy season in late March 2009, early April 2010, and early April 2011. These sampling points were timed to coincide with annual outbreaks of anthrax in the zebra population in Etosha.

We sampled only subplots for which grass treatments of *E. desvauxii* became established. Soil sampling was destructive. At each time point, we removed the PVC pipe sections (5 cm diameter by 10 cm in length) from the soil. We removed the soil from the core and retained the top 3 cm for culturing to determine the presence of *B. anthracis* in the soil. We focused on soil in the top 3 cm of the core because this soil was the most likely to come into contact with grazing animals.

We quantified *B. anthracis* total counts by culturing [19]. The top 3 cm of each soil core harvested from the experimental plots was well mixed prior to weighing and 5 g was placed in 45 ml of dilute (0.1%) sterile sodium pyrophosphate solution in a 50 ml centrifuge tube. Each tube was vortexed at maximum speed for 15 min to separate *B. anthracis* spores and cells from soil particles. After removing soil particles by a brief low speed centrifugation (300 *g* for 2 min), the supernatant was centrifuged at 3,000 *g* for 15 min to pellet spores. After removal of the supernatant, the pellet was resuspended in 5 ml 0.1% peptone water. We placed 1 ml of the resuspended mixture into a screw-capped tube and performed a dilution series (10^0^ to 10^4^) to determine the number of total viable cells in each soil sample. We spread 100 µl aliquots of each dilution onto plates made with PLET agar, which is selective for *B. anthracis* [17]. Plates were incubated at 30°C and scored on days two, three, and four. Day four counts were used in all subsequent analyses. The total counts per plate per sample were obtained by counting the plate with the dilution that produced colony forming units (CFU) between 30 and 300 per plate per sample when possible. In order to control for any differences in soil moisture, CFU/g was estimated after determining the proportion of soil moisture in each soil sample by drying it overnight at 100°C.

### Genetic analyses

We confirmed the identity and virulence markers (presence of pXO1 and pXO2 plasmids) of 5–10% of all colonies counted by performing PCR for a diagnostic chromosomal marker (BA177 [34]), lethal factor, protective antigen and capsule genes following the methods of Ramisse et al. [37]. We found that 98% of colonies were identified correctly as *B. anthracis*.

### Characterizing soil bacteria

We characterized the community of bacteria in a subset of the soil samples using the PhyloChip G2 [38] (manufactured by Affymetrix Inc., Santa Clara, CA, USA), a high-density DNA microarray to detect and monitor 8,741 bacterial and archaeal operational taxonomic units (OTUs). We used 12 PhyloChip microarrays, on three replicates of each of four treatments: spores, grass, grass + spores, and soil controls. For this part of the study, we used DNA extracted from soil samples collected during the March 2009 time point.

For the bacterial community characterization, we performed PCR with the following components per reaction: 0.02 U/µl ExTaq (Takara Bio Inc., Japan), 1× ExTaq buffer, 0.2 mM dNTP mixture, 1 µg/µl bovine serum albumin (BSA), and 300 pM each of universal bacterial primers: 27F (5′-AGAGTTTGATCCTGGCTCAG-3′) and 1492R (5′-GGTTACCTTGTTACGACTT-3′) for each genomic DNA sample. We used 10–30 ng of DNA template in a total volume of 50 µl. All samples were amplified the same time. For each sample, eight replicate PCR amplifications were performed, with a range of annealing temperatures from 48 to 58°C, with an initial denaturation at 95°C for 3 min, followed by 25 cycles of denaturation at 95°C for 30 s, annealing for 30 s, extension at 72°C for 2 min, followed by a final extension at 72°C for 10 min. Subsequently, the PCR products from the 8 reactions were combined per sample and precipitated with isopropanol using 1 µl linear acrylamide as a carrier molecule, washed twice with ice-cold 70% ethanol, and resuspended in 50 µl nuclease-free water. The pooled products were visualized using 2% agarose gels (E-gel, Invitrogen Corporation, Carlsbad, CA, USA). After gel quantification, 500 ng of the pooled PCR products for each of the 12 samples was used for the PhyloChip analysis. All samples were hybridized at the same time onto G2 PhyloChips from the same lot.

We added known concentrations of control amplicons derived from yeast and bacterial metabolic genes to 500 nanograms of pooled PCR amplicons from each soil sample. This mix was subject to fragmentation, biotin labeling, and hybridization to the G2 PhyloChip microarrays as described previously [38]. Each PhyloChip was scanned and recorded as a pixel image, and initial data acquisition and intensity determination were performed using standard Affymetrix software (GeneChip microarray analysis suite, version 5.1). Background subtraction and probe-pair scoring were performed as reported previously [38]. PhyloChip intensities were normalized according to [39]. The positive fraction (pf) was calculated for each probe set as the number of positive probe-pairs divided by the total number of probe-pairs in a probe set. An OTU was considered present if it had a positive fraction of greater than or equal to 0.9 of probes in the probe set. For each taxon/probe set, hybridization intensity (intensity) was calculated in arbitrary units using a trimmed mean (highest and lowest values were removed before averaging) of the intensities of the perfect match (PM) probes minus the intensities of their corresponding mismatch probes (MM) for all of the probe pairs in a given probe set [40]. Results for OTU intensities, presence or absence based on pf, and sample metadata are provided in Data Files S1, S2, and S3, respectively.

### Statistical analyses

All statistical tests were performed in R [41]. We used nominal logistic regression to test for an effect of spores and zebra blood on the number of experimental units where grass seeds germinated and became established or did not. We calculated confidence intervals for the binomial data on grass establishment in January 2009 using bionom.exact in the epitools library in R [42]. We used analysis of variance (ANOVA) to test for an effect of spores and zebra blood on grass height at the end of the first growing season (March 2009). Because we could only measure root length up to a maximum of 10 cm, we binned the root lengths and performed the Wilcoxin rank sum test to test for differences in root length at the end of the first growing season.

We compared the number of CFU/g detected in soil samples from each treatment at four different time points. We compared treatment effects on the rate of change in cfus/g counts over time using a linear mixed effect model (lme) [43] with our treatments (spore, grass, blood, and interactions between them) included as fixed effects and time included as a random effect. In addition, we used ANOVA to test for differences in CFU/g at the first time point (January 2009) to characterize any short term effects on that might not persist throughout the duration of the study, such as an effect of the blood treatment that could diminish with weathering.

For the soil bacterial community study, we tested for treatment effects of grass, spore, and the interaction between grass and spore treatments on number and abundance of OTUs using ANOVA. Logistic regression was used to compare the proportion of the community comprised by the different phyla. Bacterial beta-diversity metrics were calculated using FastUnifrac and Principal Coordinate Analysis (PCoA) was performed using both weighted and unweighted UniFrac distances [44]–[46].

## Results

### Establishment and growth of grass in the experimental plots

**Figure 3:**
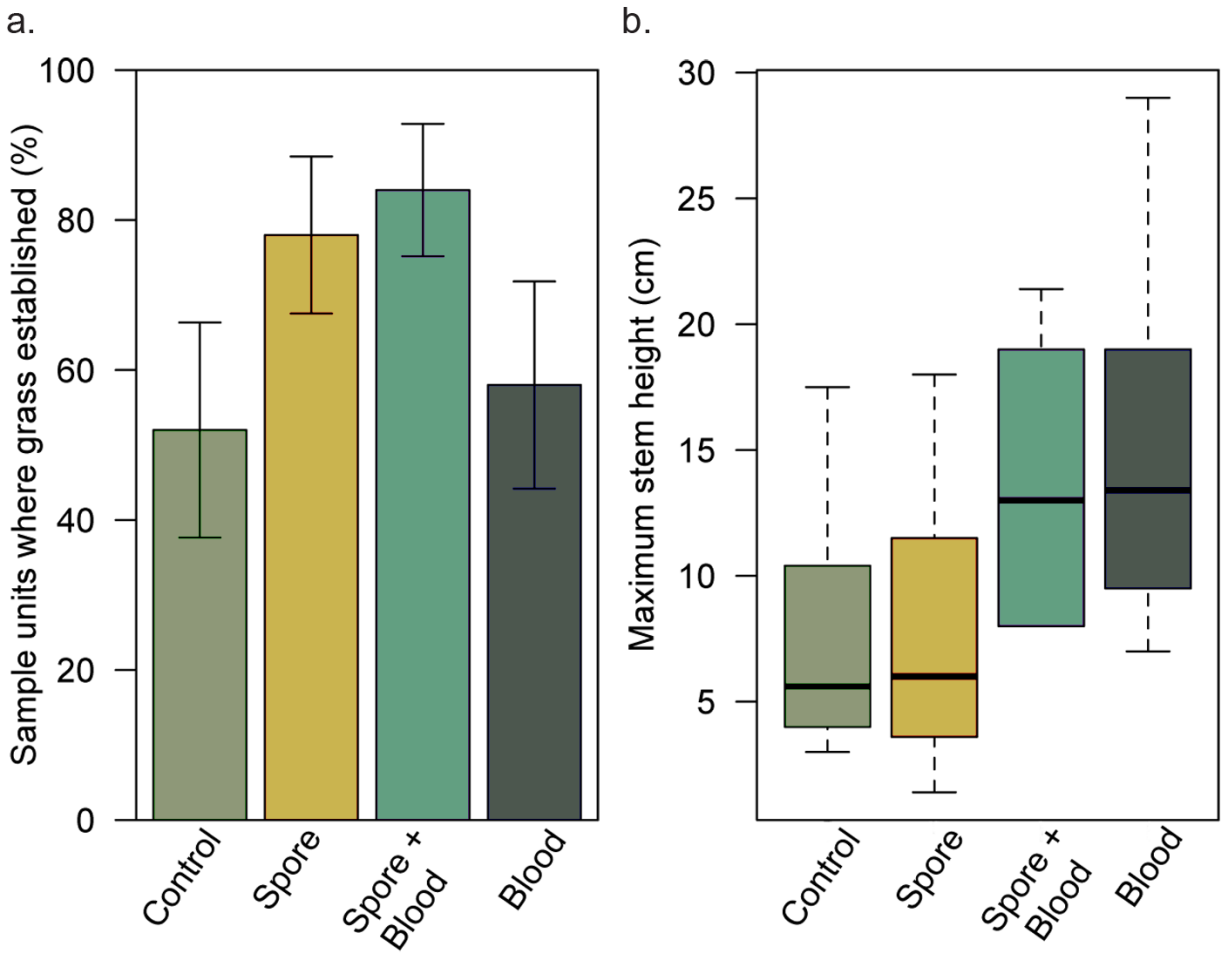
Carcass materials, such as spores and blood may promote plant growth. a. Percentage of sample units where at least one grass plant established: control (grass alone), spore, spore + blood, blood; confidence intervals are provided; N = 50 per treatment. b. Distributions of maximum stem height (cm) in the four treatments with grass present: control (grass alone), spore, spore + blood, blood; N = 9 per treatment.

Early in the growing season, we determined where grass became established in the 50 replicates per treatment that were initiated in the experiment. A grass seedling became established in 52% of the control grass treatment replicates (Figure 3a). The addition of *B. anthracis* spores increased the establishment of grass seedlings by 50% (Figure 3a; z-value = 2.673 *P* = 0.00751). The zebra blood treatment did not affect seedling establishment (Figure 3a; z-value = 0.689, *P* = 0.49) and the interaction between zebra blood and spores was not significant (z-value = 0.214, *P* = 0.83).

When we measured grass height at the end of the first growing season, we found that the addition of zebra blood increased grass height by 45% (Figure 3b; F-ratio = 9.46, DF = 1, *P* = 0.04), the addition of spores did not affect grass height (F-ratio = 0.17, DF = 1, *P* = 0.68) and the interaction between zebra blood and the spore treatment was not significant (F-ratio = 0.122, DF = 1, *P* = 0.73). Because many of the roots extended beyond 10 cm at the end of the first growing season, root lengths did not differ significantly between treatments (zebra blood: W = 125, *P* = 0.17; spore: W = 175, *P* = 0.64).

### Persistence of *B. anthracis* in soil

We found that *B. anthracis* had genetic markers for virulence throughout the duration of the experiment; multiplex PCR demonstrated that all colonies that were positive for the *B. anthracis* chromosomal marker also contained genes for lethal factor, protective antigen and capsule after eight months, 17 months, and 29 months.

**Figure 4:**
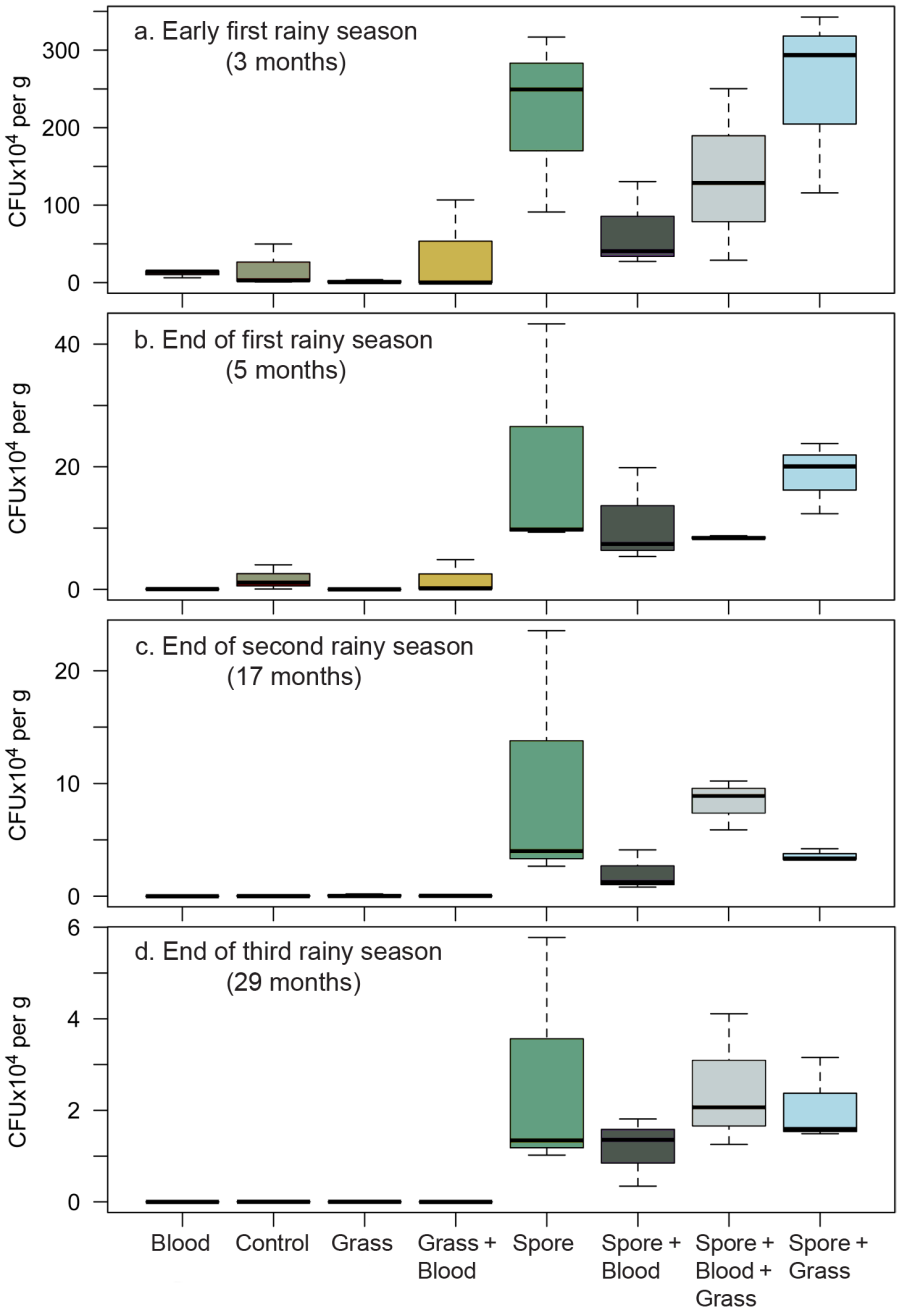
Total *B. anthracis* counts in soil (CFU ×10^4^ per g dry soil ± standard error) for eight different treatments at four different time points: a. early first rainy season (three months), b. end of first rainy season (five months), c. end of second rainy season (17 months), d. end of third rainy season (29 months). Counts are corrected for soil moisture. N = 3 per treatment.

Early in the growing season (three months), we found that there was a significant interaction between the spore and zebra blood treatments, resulting in lower CFU/g counts when both spores and blood were present (Figure 4a, spore x blood: F_1_ = 5.51, *P* = 0.032). We also detected a significant main effect of the spore treatment (Three months: Figure 4a, spore: F_1_ = 23.00, *P*<0.001). At the same time, grass did not affect the CFU/g counts (grass: F_1_ = 0.74, *P* = 0.40; spore x grass interaction: F_1_ = 0.56, *P* = 0.47).

**Figure 5:**
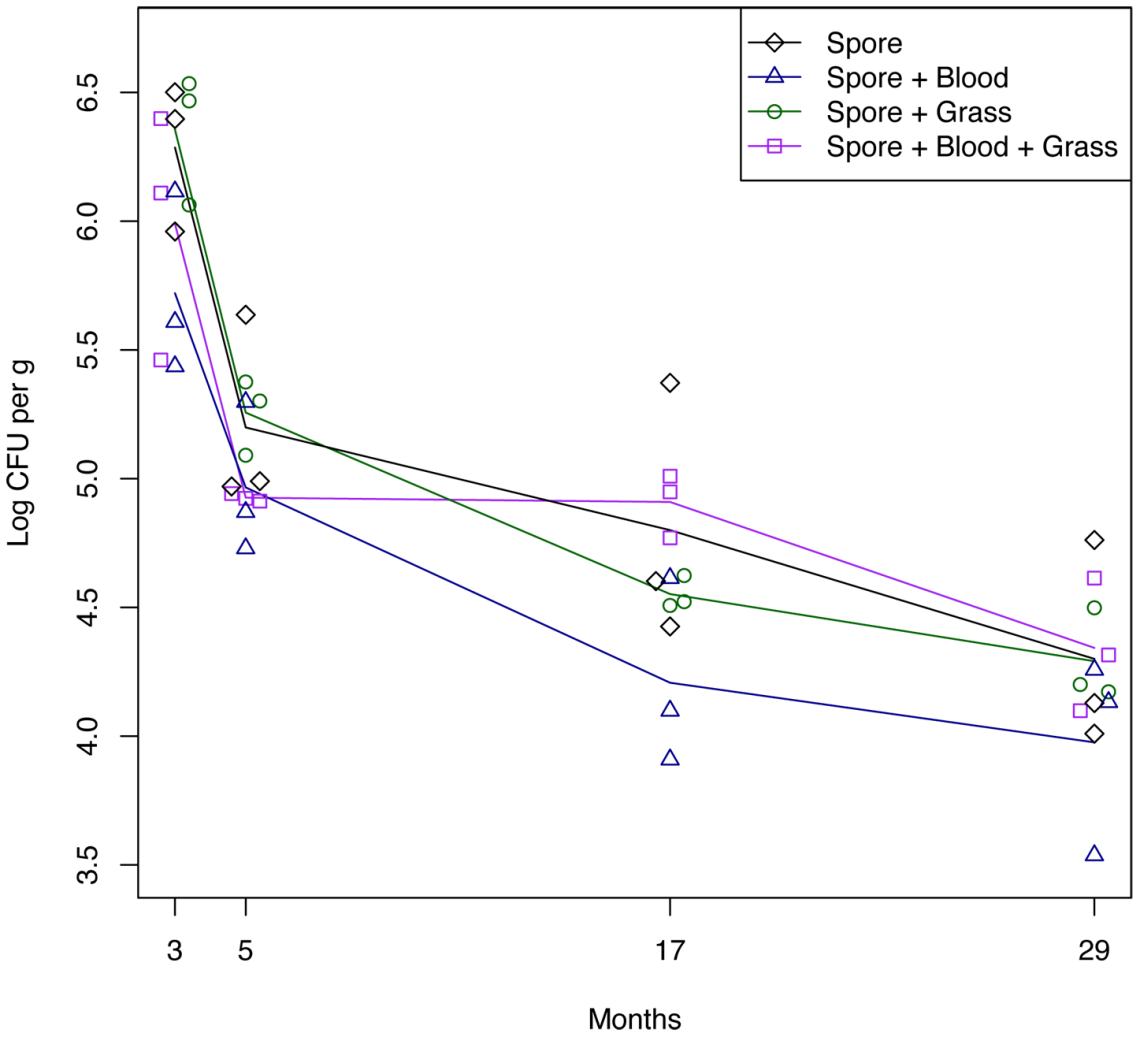
Total *B. anthracis* counts in soil (log (CFU +1) per g dry soil) for the four *B. anthracis* treatments from January 2009 until April 2011. CFU counts are indicated with open circles that are connected by a line through the mean of each of the time points. The four treatments are color coded as described in the figure legend. N = 3 per time point.

We detected a significant main effect of the spore treatment on the number of CFU/g throughout the duration of the study (Figures 4 and 5; spore, DF = 17, t-value = 4.32, *P*<0.001), which indicates that the spore treatment was effective.

Across all spore treatments, *B. anthracis* declined over time (Figure 5; month x spore: DF = 70, t-value = −3.40, *P* = 0.0011). In the treatments where spores were added, the grass treatment did not affect total *B. anthracis* counts in soil (grass x spore: DF = 17, t-value = 0.484, *P* = 0.63). However, there was a nonsignificant trend towards lower total *B. anthracis* counts in the zebra blood treatment (Figure 5; blood x spore: DF = 17, t-value = −1.72, *P* = 0.10). When we established the experiment, we placed the treatments close together in order to minimize any effects of variation in soil conditions. However, as a result of the movement of spores in flooding resulting from rainfall, we detected low levels of *B. anthracis* in some of our controls (Figure 4).

### Effects of grass and spores on bacterial community structure

**Figure 6:**
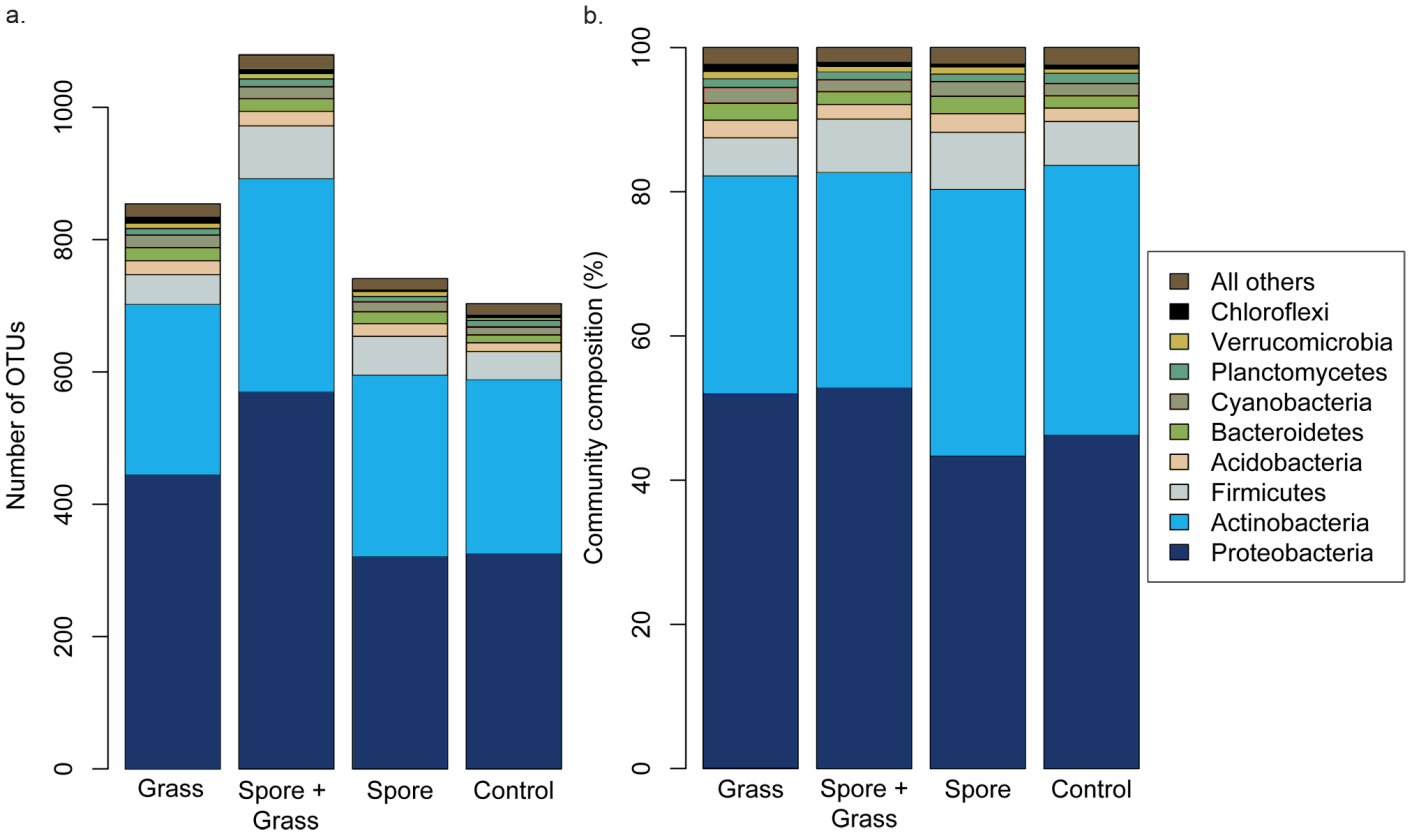
Composition of bacterial communities in bulk soil in four treatments: grass, spore + grass, spore, control (soil alone): a. Mean number of OTUs per phylum, b. Community composition (%). N = 3 per treatment.

We characterized bacterial diversity in surface soil in the experimental plots at the end of the first growing season (five months after the start of the experiment). We used the PhyloChip G2 to characterize the bacterial community in four treatments: grass, grass + spores, spores, and controls. Principal Coordinate Analysis (PCoA) on the Unifrac distance metric showed that overall the soil bacterial communities were very similar (Figure S1). After filtering the data to include only those OTUs that were present in 2 out of 3 replicates per treatment, we detected a total of 1,408 bacterial OTUs across all samples (Figure 6, Table S2). OTU richness differed significantly among the treatments; we detected 5% (38) more OTUs in the spore treatment, 16% (113) more OTUs in the grass treatment, and 53% (376) more OTUs in the spore + grass treatment compared to controls (spore: F_1_ = 10.77, *P* = 0.01, grass: F_1_ = 37.07, *P*<0.001, grass x spore: F_1_ = 5.49, *P* = 0.047). In addition to differences in number of OTUs, the proportion of OTUs per phylum differed significantly between the four treatments (Figure 6). Fourteen percent more Proteobacterial OTUs (F_1_ = 26.96, *P*<0.0001), 20% fewer Actinobacterial OTUs (F_1_ = 25.81, *P*<0.0001), and 7% fewer Verrucomicrobial OTUs (F_1_ = 21.03, *P* = 0.0017) were associated with the grass treatment (Figure 3). Thirty percent more Firmicute OTUs (F_1_ = 17.16, *P* = 0.003) and 60% more Verrucomicrobial OTUs (F_1_ = 27.21, *P*<0.001) were associated with the spore treatment (Figure 6). In addition, we detected significant interactions between the grass and spore treatments for proportion of OTUs in Acidobacteria (F_1_ = 14.55, *P* = 0.005), Bacteroidetes (F_1_ = 9.20, *P* = 0.016), Cyanobacteria (F_1_ = 8.18, *P* = 0.02), and Verrucomicrobia (F_1_ = 12.72, *P*<0.01). After correcting for multiple comparisons, we did not detect significant differences among the treatments in abundance of individual OTUs.

## Discussion

Inspired by recent laboratory findings [5], [6], [8] that suggest that *B. anthracis* may have activity outside of a vertebrate host, we explored interactions between a native grass and *B. anthracis* in an experimental field enclosure. We tested the hypothesis that carcass materials, such as *B. anthracis* and zebra blood, might increase grass growth. We found that the addition of *B. anthracis* spores to soil containing *E. desvauxii* seeds substantially increased its rate of establishment. In addition, we found that the addition of zebra blood to soil increased grass height. Prior studies in North America showed increased plant biomass at carcass sites in response to nutrient deposition from a carcass [21]–[23]. Thus we hypothesized that the zebra blood might fertilize the grass. Nonetheless we were surprised to find that the treatment of *B. anthracis* spores alone (without the nutrient addition that can be expected in the blood treatment) affected the rate of grass seedling establishment in our experimental plots.

In fact, the observation that *B. anthracis* may have significant interactions with plants is not unexpected. Many *Bacillus* species are known plant-growth promoting bacteria that exert beneficial effects on plant growth and development [47], [48]. Various strains of *B. cereus* have been introduced into soils, onto seeds, roots, tubers, and other planting materials in efforts to improve the growth of crops [49]. In addition to facilitating nutrient uptake and supplying growth-promoting substances to plant roots [50], [51], plant-growth promoting bacteria can also induce systemic resistance, providing protection from plant pathogens and insects [52], and can improve drought tolerance [24]–[26].

We hypothesized that the presence of grass might reduce rate of decay of *B. anthracis* in soil if there was replication in the rhizosphere or if the grass reduced the effects of weathering. While plants may provide a reservoir that promotes persistence of *B. thuringiensis* and *B. anthracis* in the environment [5], [53], in concordance with previous studies, we found no evidence for multiplication or increased persistence of *B. anthracis* in soil cores with *E. desvauxii*. Instead of providing an incubator for multiplication in the environment, the association with plants for *B. anthracis* and *B. thuringiensis* may be driven at least in part by increased likelihood of contact with herbivorous hosts [54]. However, when the grass was newly established in the experimental plots (three months), we found that the addition of blood to the spore treatments significantly lowered the *B. anthracis* CFU/g counts. Some of the *B. anthracis* spores may have germinated in the soil in response to the nutrients from the blood and subsequently failed to replicate. Prior studies show that *B. anthracis* spores germinate in water or soil augmented with viscera or blood from animals [3], [4], [55]. Vegetative cells have high nutrient requirements and may be unable to thrive outside a host without nutrient augmentation [55].

**Figure 7:**
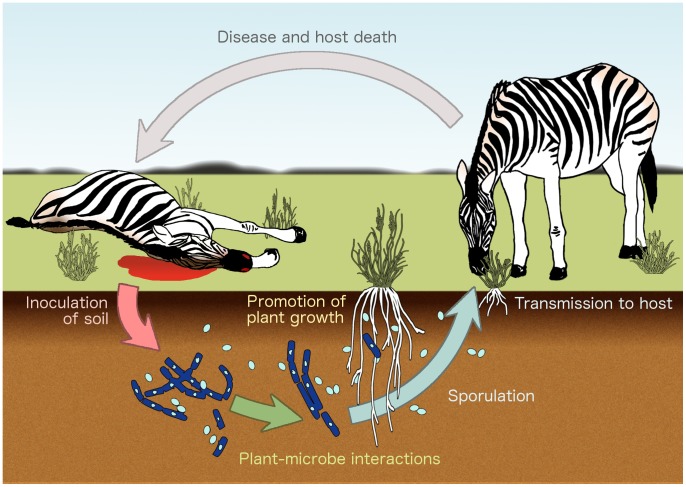
An ingestion-transmission cycle of *B. anthracis*. Many anthrax infections occur in herbivorous animals, such as zebra, that consume infectious spores while grazing or browsing [17], [29], [30]. Spores germinate within the host to produce vegetative cells that cause a fulminant infection. After host death, terminal hemorrhaging and scavenging releases blood, body fluids, and vegetative *B. anthracis* cells that inoculate soil in the surrounding area [17], [69], [70]. While it has long been held that these vegetative cells cannot survive competition with soil-dwelling bacteria and must sporulate rapidly [3], [4], [55], recent studies indicate the potential for *B. anthracis* to form biofilms [6], [71] and to persist in the rhizosphere [5]. In addition to the nutrients found in carcass materials, *B. anthracis* inoculation appears to promote plant growth, which may attract grazing hosts and increase the rate of anthrax transmission. Illustration by Kelsey Wood.

These findings suggest that naturally occurring anthrax carcass sites may exhibit increased plant establishment and productivity that potentially attracts grazing hosts to these infectious sites beyond that expected for uninfected carcass sites (Figure 7). In future studies, we will compare vegetation growth and host use of carcass sites that differ in the presence of *B. anthracis*. Herbivores selectively forage on vegetation in productive, nutrient rich soils in a heterogeneous landscape [56] and the carcass site is a primary source of environmental transmission of *B. anthracis* to potential hosts. In his seminal research on anthrax in the late 19^th^ century, Louis Pasteur identified carcass sites as a key area for anthrax transmission [15] and demonstrated that grazing animals can acquire anthrax infections by ingestion of abrasive plants inoculated with *B. anthracis* [15]. Grazing hosts may come into contact with pathogens by ingesting soil along with the leaves and roots of plants [30] and pathogens may colonize the interior of plants [57]. Rain splash onto plants can provide a potential transmission route for pathogens in soil, such as *Salmonella enterica* serovar Typhimurium [58]. Here we did not find detectable amounts of *B. anthracis* on *E. desvauxii* leaves. However, this may reflect our experimental design in which spores were narrowly deposited within a 5 cm diameter and not widely spread out as would be expected at a carcass site. In addition to occurring on the leaf exterior, *Salmonella enterica* serovar Typhimurium successfully invades the interior of tomato plants, providing another potential transmission route [57]. Determining whether *B. anthracis* also colonizes the interior of plants would require similarly careful laboratory and greenhouse experiments.

Finally we tested whether *E. desvauxii* or *B. anthracis* affects the structure of bacterial communities occurring in bulk soil. Overall the bacterial communities were very similar; however, we detected significant effects of *E. desvauxii* and *B. anthracis* on OTU richness and bacterial community composition. Previously Saile and Koehler [5] showed in the laboratory that *B. anthracis* germinates in the grass rhizosphere. It may be that the significant interaction that we detected between the spore and grass treatments on overall OTU richness reflects some activity of *B. anthracis* in association with *E. desvauxii*. In addition, community composition in bulk soil associated with *E. desvauxii* and *B. anthracis* differed from controls. Bulk soil associated with *E. desvauxii* contained a greater number and proportion of Proteobacteria, while bulk soil associated with *B. anthracis* contained a greater number and proportion of Firmicutes. While the effect of plants on bulk soil is less well studied, plants are known to affect the composition and relative abundance of bacterial populations in the rhizosphere [59]–[63]. The observation that Proteobacteria comprise a greater number and proportion of bacteria in bulk soil associated with *E. desvauxii* is expected given that Proteobacteria are dominant members of rhizosphere communities [59].

Despite the central role of the environment in its transmission, little is known about the survival and activity of *B. anthracis* outside of a host, due in part to a lack of manipulative field experiments. Van Ness [64] proposed that under conditions of alkaline pH, high soil moisture, and the presence of organic matter, *B. anthracis* maintains incubator areas where it multiplies in the environment but he failed to provide empirical support for his hypothesis. Field observations of carcass sites suggest that *B. anthracis* might exhibit at least some limited level of activity in the environment, including the loss of one or both of virulence plasmids over time [65], [66], the acquisition of antibiotic resistance [67], and the maintenance of undiminished soil contamination levels for years despite exposure to the elements [17]. Moreover, several studies suggest that *B. anthracis* may have the ability to interact with other members of the grassland-soil community [5]–[8], [15], [16]. Schuch and Fischetti [6] showed that bacteriophages provide *B. anthracis* with alternatives to sporulation outside of the vertebrate host. The *B. anthracis* isolate studied here was subsequently determined to contain a novel lysogenic bacteriophage that was recently characterized [68]. Future studies will explore the role that such phages may play in the interaction between *B. anthracis* isolates and plants. In conclusion, the findings presented here suggest that *B. anthracis* interacts with the grass, *E. desvauxii* in the field, promoting its establishment, and providing a potential disease transmission route to grazing hosts.

## Supporting Information

Figure S1 Principal Coordinate Analysis (PCoA) of unweighted (a.) and weighted (b.) UniFrac distances. Four treatments are indicated by color in the figure as follows, green squares: control (no grass + no spores), red triangles: grass, blue triangles: spores + grass, black circles: spores.
(PDF)

Data File S1 PhyloChip Operational Taxonomic Units (OTUs) intensities.
(CSV)

Data File S2 PhyloChip binary data for OTU presence or absence.
(CSV)

Data File S3 PhyloChip OTU metadata.
(CSV)

Table S1 Physical and chemical properties of five soil samples from the experimental field enclosure in Etosha National Park, Namibia.
(DOCX)

Table S2 Number of bacterial OTUs detected by the PhyloChip G2 in bulk soil for four treatments: grass, spore + grass, spore and control (soil alone).
(DOCX)
