# Supplementary figures and images for: Interactions between Bacillus anthracis and Plants May Promote Anthrax Transmission
Source: PLoS Negl Trop Dis. 2014 Jun 5;8(6):e2903. doi: 10.1371/journal.pntd.0002903 (PMC4046938; doi:10.1371/journal.pntd.0002903)

a.

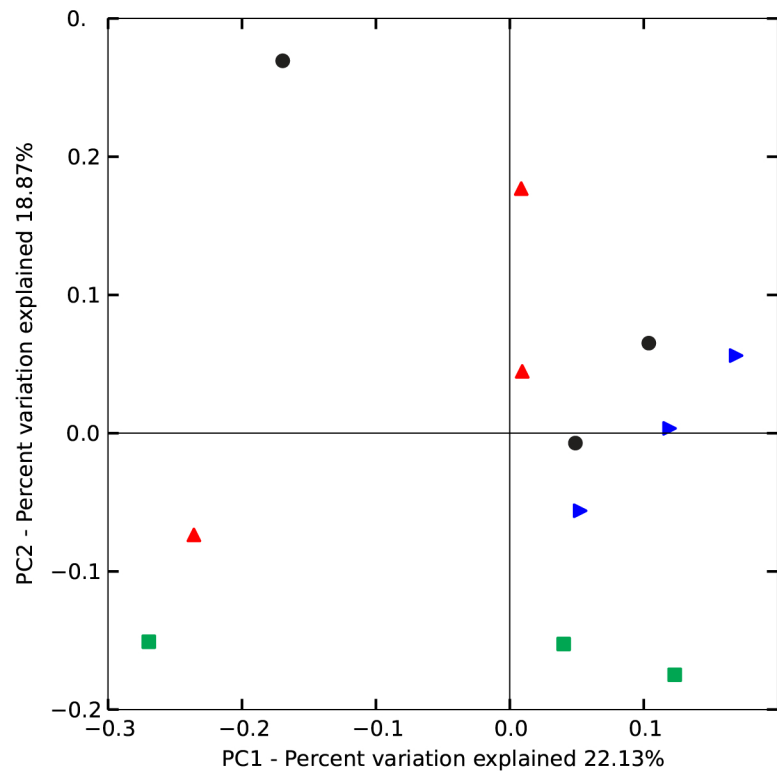

b.

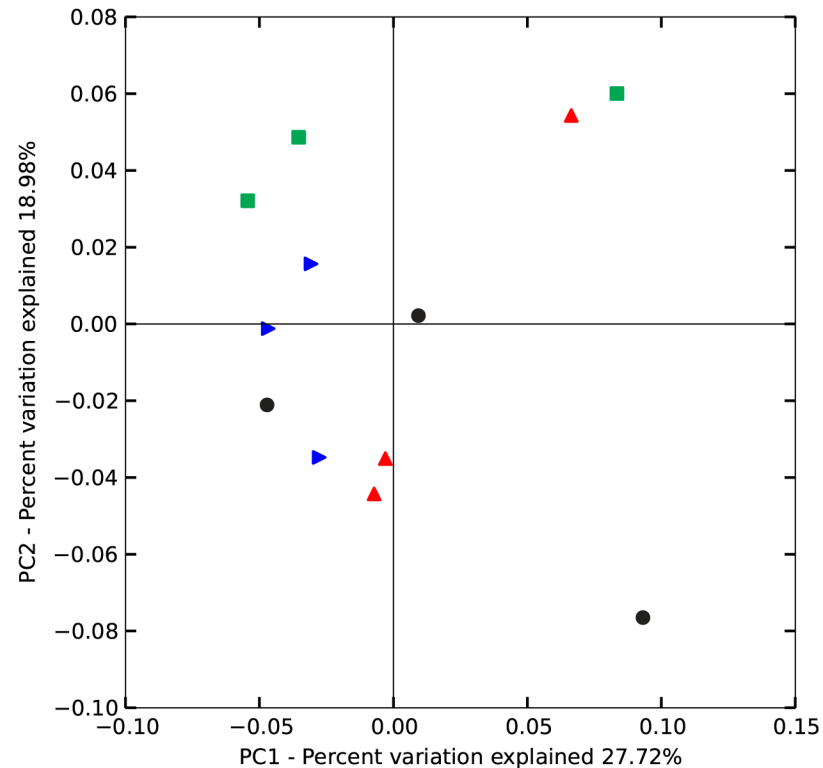

Supplement: Figure S1 — Principal Coordinate Analysis (PCoA) of unweighted (a.) and weighted (b.) UniFrac distances. Four treatments are indicated by color in the figure as follows, green squares: control (no grass + no spores), red triangles: grass, blue triangles: spores + grass, black circles: spores. (PDF) [file pntd.0002903.s001.pdf]
