# Supplementary material for: Interactions between Bacillus anthracis and Plants May Promote Anthrax Transmission
Source: PLoS Negl Trop Dis. 2014 Jun 5;8(6):e2903. doi: 10.1371/journal.pntd.0002903 (PMC4046938; doi:10.1371/journal.pntd.0002903)
Supplement: Table S1 — Physical and chemical properties of five soil samples from the experimental field enclosure in Etosha National Park, Namibia. (DOCX) [file pntd.0002903.s005.docx]

Table S1. Physical and chemical properties of five soil samples from the experimental field enclosure in Etosha National Park, Namibia. Electrical conductivity is abbreviated as EC and organic matter is abbreviated as OM.

| Sample | P ppm | K  ppm | Ca  ppm | Mg ppm | Na ppm | pH | EC  uS/cm | OM  % | CaCO_3_ % | Texture | Sand % | Clay  % | Silt  % |
| --- | --- | --- | --- | --- | --- | --- | --- | --- | --- | --- | --- | --- | --- |
| 1 | 3.32 | 512 | 3874 | 246 | 1334 | 7.69 | 2100 | 2.18 | 5-10 | sandy loam | 54.6 | 8 | 37.5 |
| 2 | 3.93 | 570 | 3604 | 234 | 263 | 7.80 | 245 | 2.03 | 5-10 | sandy clay loam | 58.6 | 22.4 | 18.9 |
| 3 | 2.92 | 554 | 3718 | 240 | 730 | 7.74 | 1350 | 2.69 | 5-10 | sandy loam | 58.5 | 7.2 | 34.3 |
| 4 | 2.17 | 452 | 3774 | 240 | 259 | 7.93 | 290 | 1.95 | 5-10 | sandy clay | 51.8 | 35.6 | 12.6 |
| 5 | 3.05 | 398 | 3856 | 234 | 101 | 7.86 | 140 | 2.79 | 5-10 | sandy loam | 58 | 10.3 | 31.7 |
| Mean | 3.08 | 497 | 3765 | 239 | 537 | 7.80 | 825 | 2.33 | 5-10 |  | 56.3 | 16.7 | 27.0 |
| Std Error | 0.29 | 32.1 | 49.2 | 2.24 | 225 | 0.04 | 387 | 0.17 |  |  | 1.34 | 5.46 | 4.79 |
