# Supplementary material for: Interactions between Bacillus anthracis and Plants May Promote Anthrax Transmission
Source: PLoS Negl Trop Dis. 2014 Jun 5;8(6):e2903. doi: 10.1371/journal.pntd.0002903 (PMC4046938; doi:10.1371/journal.pntd.0002903)
Supplement: Table S2 — Number of bacterial OTUs detected by the PhyloChip G2 in bulk soil for four treatments: grass, spore + grass, spore and control (soil alone). (DOCX) [file pntd.0002903.s006.docx]

Table S2. Number of bacterial OTUs detected by the PhyloChip G2 in bulk soil for four treatments: grass, spore + grass, spore and control (soil alone).

|  |  | **Treatment** | | | | | | | |
| --- | --- | --- | --- | --- | --- | --- | --- | --- | --- |
|  |  | **Grass** | | **Spore + Grass** | | **Spore** | | **Control** | |
| **Domain** | **Phylum** | **mean** | **SE** | **mean** | **SE** | **mean** | **SE** | **mean** | **SE** |
| Bacteria | Total | 854 | 51.4 | 1079 | 24.1 | 741 | 34.3 | 703 | 45.3 |
|  | Proteobacteria | 444 | 40.0 | 570 | 26.8 | 321 | 25.2 | 325 | 23.6 |
|  | Actinobacteria | 258 | 6.9 | 322 | 14.9 | 274 | 7.9 | 263 | 15.0 |
|  | Firmicutes | 45 | 6.2 | 80 | 6.8 | 59 | 4.18 | 43 | 2.4 |
|  | Acidobacteria | 21 | 0.58 | 22 | 1.20 | 19 | 0.58 | 13 | 1.67 |
|  | Bacteroidetes | 20 | 2.19 | 19 | 2.65 | 18 | 1.00 | 12 | 1.00 |
|  | Cyanobacteria | 19 | 1.53 | 18 | 1.33 | 15 | 0.58 | 12 | 0.88 |
|  | Planctomycetes | 10 | 0.882 | 12 | 0.58 | 8 | 0.58 | 10 | 2.00 |
|  | Verrucomicrobia | 8 | 1.67 | 8 | 0.88 | 7 | 1.33 | 4 | 0.00 |
|  | Chloroflexi | 9 | 0.67 | 6 | 0.67 | 3 | 0.67 | 4 | 0.58 |

*Note:* Phyla with fewer than five OTUs in at least one treatment group are excluded.
